# The Treatment of Backward Displacements of the Uterus

**Published:** 1907-08-10

**Authors:** 


### Gynæcology.

### THE TREATMENT OF BACKWARD DISPLACEMENTS OF THE UTERUS.

Retroversion and retroflexion of the uterus, perhaps the commonest lesion of the female pelvic organs which comes before us, often requires much discretion in its treatment, which is only too frequently extremely disappointing to the patient in its results. It is so easy to put in a pessary when a backward displacement is found that it is not surprising to find that a large proportion of such cases are thus treated. If, however, a careful examination is made of the numberless people who are wearing pessaries, it will be found that the displacement is corrected in a very small proportion. More important still, a few questions put to these people will elicit the fact that a still smaller proportion of them have received any relief whatever for the symptoms they originally complained of. These two points are brought out very clearly in all gynæcological out-patient practice in hospitals, because so many of the patients seen have been treated before either at other hospitals or by private practitioners. Now it must be clearly remembered that the use of a pessary has two distinct objects in view, the one to correct the displacement found, and the other to relieve the symptoms complained of. There can be no doubt which of these is the more important to the patient, for it makes no difference to any woman whether her uterus is anteverted or retroverted, provided the displacement gives rise to no symptoms. For this reason, then, we have to consider very carefully before we attempt to correct a backward displacement by a pessary, whether by this means we shall cure the symptoms. This is sometimes difficult, sometimes impossible, which, however, is no excuse for indiscriminate pessary treatment in these cases. It is possible in many cases, by a careful consideration of the symptoms complained of, to arrive at some conclusion, whether these symptoms are really caused by the displacement or not, and therefore whether they will be relieved by the correction of the displacement.

The points to be considered before a decision can be arrived at are these:—

1. Marriage and pregnancy.
2. Menstrual history.
3. Tenderness and descent of the ovaries.
4. Aches and pains.
5. Discharges.
6. Bladder and rectal symptoms.
7. Obscure reflex symptoms.

As pregnancy, labour and abortion are the commonest, and practically the only common causes of acquired backward displacements, we have here at once a dividing line between two great groups of cases. Those cases in which pregnancy has not occurred are with very few exceptions congenital, those in which pregnancy has occurred are often acquired, although we must not forget that pregnancy in a congenitally retroverted uterus occurs sufficiently often to be regarded as no rarity. It is a matter of common experience that a congenitally retroverted uterus gives rise to no symptoms *per se*, but when symptoms are associated with it there must have been a pregnancy in or an infection of the organ. Also an acquired displacement, the result of labour or abortion, may have no associated symptoms, provided there is no congestion.

If menstruation is normal in quantity and not painful we have clear evidence that there is no uterine congestion, but if the reverse is the case uterine congestion is certainly present, and may be either the result of bacterial infection or may be actually caused by some interference with the circulation by the backwardly directed fundus.

Tender prolapsed ovaries when present are perhaps the most important accompanying condition of a backward displacement. If these organs can be raised out of Douglas' pouch by correcting the displacement, in some cases the congestion which causes the tenderness may be relieved by appropriate treatment, and after a time pessaries can in some cases be used. As a rule, however, this condition calls for some operative treatment.

Backache is a prominent feature in many of these cases, and that this is sometimes caused by the actual displacement is proved by the fact that occasionally correction of the same relieves the pain. On the other hand, backache is a prominent symptom of uterine congestion, and if the latter is caused by infection the pain will not be relieved by correcting the displacement. It must not be forgotten, however, that backache is the common lot of most women, and is dependent on many things quite apart from the uterus. It is only necessary to mention dyspepsia, lithiasis, myalgia, renal diseases, spinal diseases, neurasthenia, etc., to realise this. In fact, in practice it is found that a very small percentage of backaches are cured by correcting a backward displacement of the uterus.

What has been said of menstruation applies equally well to discharges; these depend on congestion of either the cervix or the body of the uterus or both, and are only set up by displacements when these actually cause uterine congestion. Discharges are more commonly the result of bacterial infection.

That the bladder is sometimes interfered with by retroversions is a fact, but the retroverted gravid uterus is the only common cause of retention of urine. The unimpregnated retroverted uterus but rarely causes any bladder disturbance, never retention, only occasionally frequency of micturition. The unimpregnated retroverted uterus never causes constipation or hæmorrhoids. This statement can be proved by anyone who treats these cases; constipation and hæmorrhoids are never cured by the correction of a displaced uterus. That most of these subjects have constipation is true, but so do the majority of women who have no displacements.

Backward displacements in virgins are usually congenital, cause no symptoms, and therefore should not be treated by pessaries with very few exceptions. There is nothing worse for the well-being of an unmarried woman than to have her attention directed towards her sexual organs by the use of a pessary.

